# How does teacher support impact the academic achievement of boarding junior high school students? The mediating role of academic self-concept and the moderating effect of academic anxiety

**DOI:** 10.3389/fpsyg.2026.1786081

**Published:** 2026-04-28

**Authors:** Han Xue, Zirui Yang, Jianwen Guo

**Affiliations:** 1 School of Education, Minzu University of China, Beijing, China; 2 College of Elementary Education, Capital Normal University, Beijing, China

**Keywords:** academic achievement, academic anxiety, academic self-concept, boarding junior high school students, teacher support

## Abstract

**Aim:**

This study aimed to examine the mediating role of academic self-concept in the relationship between teacher support and academic achievement, as well as the moderating role of academic anxiety in the relationships between teacher support and both academic self-concept and academic achievement.

**Sample:**

This cross-sectional study involved 2,239 boarding junior high school students (1,132 boys, 1,161 girls; M_age_ = 13.33, SD = 0.94), recruited via convenience sampling from Yunnan and Zhejiang provinces, China. Inclusion criteria were as follows: (1) be officially enrolled as junior high school students; (2) boarding students are those who stay overnight at school every day except for major holidays; and (3) provide voluntary consent to participate.

**Methods:**

Teacher support was measured using the Perceived Teacher Support subscale of the School Climate Scale; academic self-concept was assessed with the General Academic Self-Concept Scale; academic achievement was indexed by students’ self-reported class rank; and academic anxiety was evaluated using the Academic Anxiety subscale of the Academic Emotions Questionnaire.

**Results:**

The findings revealed that academic self-concept and it’s four dimensions mediated the relationship between teacher support and academic achievement (*β* = 0.12, 0.08, 0.07, 0.10, 0.06, respectively). Additionally, academic anxiety moderated the relationships between teacher support and academic anxiety on academic self-concept (*β* = −0.03) and academic achievement (*β* = −0.04), moreover, academic anxiety moderated the relationships between teacher support and the value of academic achievement dimension of academic self-concept (*β* = −0.10), as well as teacher support and academic achievement when mediator is self-control of academic behavior (*β* = −0.05), perception of academic ability (*β* = −0.05) and experience of academic emotion (*β* = −0.04).

**Conclusion:**

Although the sample of this study comes from boarding schools in specific provinces of China, which may limit the generalizability of the findings, this study highlights the significant impact of teacher support on academic achievement and underscores the importance of academic self-concept and academic anxiety in influencing these outcomes. This enhances our understanding and offers insights for re-examining classic theories such as social cognitive theory and cognitive resource theory.

## 1 Introduction

Academic achievement is often viewed as a yardstick of students’ knowledge attainment and their adjustment to school, and thus, it has long been in the spotlight of scholarly research (Tao et al., 2022). It carries significant positive implications; for example, students who perform well academically tend to develop positive beliefs about work and life satisfaction (Crede et al., 2015). Previous studies have identified several factors influencing academic achievement, including parental academic support such as involvement and encouragement (Choe, 2020). Additionally, a supportive family environment and higher socioeconomic status are also crucial for academic success. Specifically, a positive family environment and higher socioeconomic status provide better resources and support, leading to improved academic outcomes (Jiang et al., 2023; Wang and Sheikh-Khalil, 2021). There is no doubt that teachers are impactful agents in students’ educational pursuits (Kim et al., 2017), as academic performance is highly valued and teachers are regarded as knowledge authorities in Chinese culture. Teachers’ most direct influence on students is through the support they provide, which is particularly crucial for boarding students. Many studies supported the point, for instance, Pfeiffer et al. (2016) found that, compared with day school students, boarding school students perceived more social support from their teachers but less from their parents. Additionally, Ma'arif and Rofiq (2018), Wang and Zhang (2020) further demonstrated that teacher support plays a key and unique role to students in boarding schools. Boarding students may exhibit relatively lower academic performance than day students in junior high schools, as supported by empirical evidence from China, highlighting the importance of examining factors that influence boarders’ academic achievement (Wu, 2023).

The importance of positive teacher support for variables associated with students’ academic performance or their other experiences in school, such as academic self-efficacy (Kang et al., 2024), academic engagement (Tao et al., 2022), academic resilience (Jannah et al., 2024), academic expectancy, self-esteem, and depressive symptoms (Reddy et al., 2003) has been well-documented in a substantial body of literature. In fact, according to the ecology theory of human development (Bronfenbrenner, 1979), teacher support can serve as a proximate factor influencing all aspects of academic performance among boarding junior high school students. To this end, this study will explore the relationship between teacher support and academic achievement and the mechanisms underlying this relationship among boarding junior high school students. However, several gaps remain. Existing studies have primarily focused on the direct effects of teacher support, with limited attention to the underlying mechanisms linking teacher support to academic achievement. Moreover, few studies have integrated mediating and moderating processes within a single framework, particularly among boarding junior high school students. Therefore, this study aims to examine the mediating role of academic self-concept and the moderating role of academic anxiety in this relationship. Theoretically, this study contributes to the literature by providing a more comprehensive understanding of how and under what conditions teacher support influences academic achievement, particularly within the context of boarding junior high school students.

### 1.1 Teacher support and academic achievement

Although the operational definitions, dimensions, and measurements of teacher support vary across studies (Tao et al., 2022), it typically refers to a combination of emotional and academic support, with a focus on social support from teachers in the study (Jia et al., 2009). Many previous studies have found a positive relationship between teacher support and academic achievement. For example, Tao et al. (2022) noted that teacher support benefits students’ learning outcomes, particularly in high-stakes testing and accountability contexts. Affuso et al. (2022) further confirmed that teachers help students better understand their capabilities by providing concrete support in tasks and assigning marks, thereby guiding students in refining their goals and achieving satisfactory results more easily. For boarding students, these relationships are also supported. Xu (2019) indicated that the student-teacher relationship is a positive predictor of academic achievement for boarders. Recent studies emphasize the importance of teacher support in both emotional and academic domains. Emotional support, such as empathy and encouragement, enhances students’ well-being and motivation (Skaalvik and Skaalvik, 2020). Meanwhile, academic support, including tailored feedback and additional assistance, directly improves academic performance (Zheng et al., 2022). For boarding students, who may encounter unique challenges, these forms of support are especially vital.

To this end, this study posits that teacher support significantly and positively predicts the academic achievement of boarding junior high school students and aims to explore the mechanisms underlying this relationship within this group.

### 1.2 The mediating effect of academic self-concept

Academic self-concept is defined as students’ stable perceptions and evaluations of their strengths, abilities, and knowledge in academic areas, which mainly consists of four factors: perceived academic competence, academic behavioral self-regulation, enjoyment of academic experience and value of academic achievement (Guo et al., 2011). Many scholars believed that there is positive relationship between teacher support and academic self-concept. For example, Zhao and Yang (2022) believed that teacher support significantly affected academic self-concept among Chinese students. This is further supported by Wu and Kang (2023), who emphasized the critical role of teacher support in shaping students’ perceptions of their academic abilities. This influence is particularly vital for boarding students, who often depend more on their teachers for both academic guidance and emotional support. Existing research also supports the influence of teacher support on academic self-concept at the specific dimension level. For example, when students perceived more teacher support, they were willing to spend more time on their learning (Zhao and Yang 2022) and tend to persevere more (Kang et al., 2024). Additionally, students benefit from the essential structure that teachers provide by managing activities and offering feedback, which not only enhances their perceived academic competence (Affuso et al., 2022) and confidence in their abilities, but also fosters greater enjoyment of their academic experiences and increases the value they place on their achievements (Wu and Kang, 2023).

Regarding the relationship between academic self-concept and academic achievement, self-enhancement theorists argued that self-concept variables are primarily cause of academic achievement (Calsyn and Kenny, 1977), and the longitudinal and cross-sectional correlations indicated that academic self-concept was more strongly correlated with academic achievement (Guay et al., 2010). Existing research supports the influence of academic self-concept on academic achievement across various dimensions. For example, Zimmerman (2008) highlights that self-regulation behaviors improve academic performance, while Marsh and Craven (2006) show that perceived competence strongly predicts academic success. Additionally, Patrick et al. (2007) emphasize the role of emotional support in enhancing engagement and achievement, and Elliot and Dweck (2005) link achievement motivation to higher academic outcomes. These studies collectively demonstrate the significant impact of specific aspects of self-concept on academic performance.

Based on previous studies, we propose the first hypothesis:

*H1*: Academic self-concept and its four dimensions (perceived academic competence, academic behavioral self-regulation, enjoyment of academic experience, and value of academic achievement) serve as mediators between teacher support and academic achievement. Specifically, when boarding middle school students receive more teacher support, they will develop a higher academic self-concept, which in turn will further enhance their academic performance.

### 1.3 The moderating effect of academic anxiety

Academic anxiety refers to an emotional response among students during their educational journey, marked by concerns about how their perceived or anticipated outcomes may undermine their self-esteem and sense of value (Cheng, 1998). Previous research suggests that any single identity attribute within the study group can potentially contribute to heightened academic anxiety. For instance, from the perspective of boarding, Xu (2019) used mathematics as an example and uncovered that boarders exhibit significantly higher levels of math anxiety compared to their non-boarder peers. Mander and Lester (2017) further discovered that boarding students reported significantly higher levels of anxiety and stress at the end of Grade 8 compared to non-boarding students after incorporating the factor of time. From the viewpoint of junior high school, academic anxiety emerges as the predominant negative emotional response throughout the academic experience (Cheng, 1998). Students encounter pressures associated with “academic progression and employment” and endure extended periods characterized by competition, tension, and anxiety, frequently resulting in learning anxiety issues during this phase (Cheng, 1998). Moreover, from a cultural standpoint, previous studies indicated that students typically experience heightened levels of academic anxiety in China (Frenzel et al., 2007), where academic achievement is highly esteemed as well as task importance and achievement pressure are related to anxiety (Goetz et al., 2013). Subjective values of these activities and outcomes, the second dimension of the control-value theory, also address this point (Pekrun, 2006).

In the context of anxiety’s impact on academic self-concept and achievement, while most existing research indicates a substantial negative correlation between anxiety and both factors (e.g., Brumariu et al., 2023; Cheng, 1998; Desideri et al., 2019; Putwain et al., 2020), there are also studies that adopt a more cautious perspective on this issue. For instance, Brumariu et al. (2023) pointed out that anxiety in itself might not be a hindrance to some academic outcomes and does not strongly hinder academic achievement. The reason for this outcome may stem from the complexity inherent in anxiety itself. According to control-value theory, the general functional mechanisms of human emotions are bound to universal, species-specific characteristics of our mind. In contrast, the specific content of emotions, as well as specific values of process parameters such as the intensity of emotions, may vary across different cultures, genders, and individuals (Pekrun, 2006). This suggests a necessity for further detailed analysis of anxiety; thus, academic anxiety was categorized into low and high levels for examination in this study.

Previous research indicates a robust negative correlation between anxiety and social support. Specifically, individuals with low anxiety tend to experience significantly higher levels of social support compared to those with high anxiety (Li et al., 2021). Additionally, anxiety to some extent affects learners’ acceptance of feedback. Generally, learners with low anxiety demonstrate higher acceptance and experience more beneficial effects from feedback compared to those with high anxiety (Li and Wang, 2018). From this, we can infer that boarding junior high school students with low anxiety are more likely to receive teacher support, actively implement teacher suggestions, and consequently are more likely to develop positive academic self-concepts and achieve higher academic achievement. In contrast, boarding junior high school students with high anxiety may encounter challenges in obtaining teacher support and exhibit lower acceptance of academic advice, thereby diminishing the impact of teacher support on the fostering of positive academic self-concepts and academic achievement within this group.

Based on previous studies, we propose the following hypotheses:

*H2.1*: Academic anxiety may moderate the effect of teacher support on academic self-concept. Specifically, this relationship weakens for boarding junior high school students with high academic anxiety compared to those with low academic anxiety.

*H2.2*: Academic anxiety may moderate the effect of teacher support on academic achievement. Specifically, this relationship weakens for boarding junior high school students with high academic anxiety compared to those with low academic anxiety.

### 1.4 The present study

**Figure 1:**
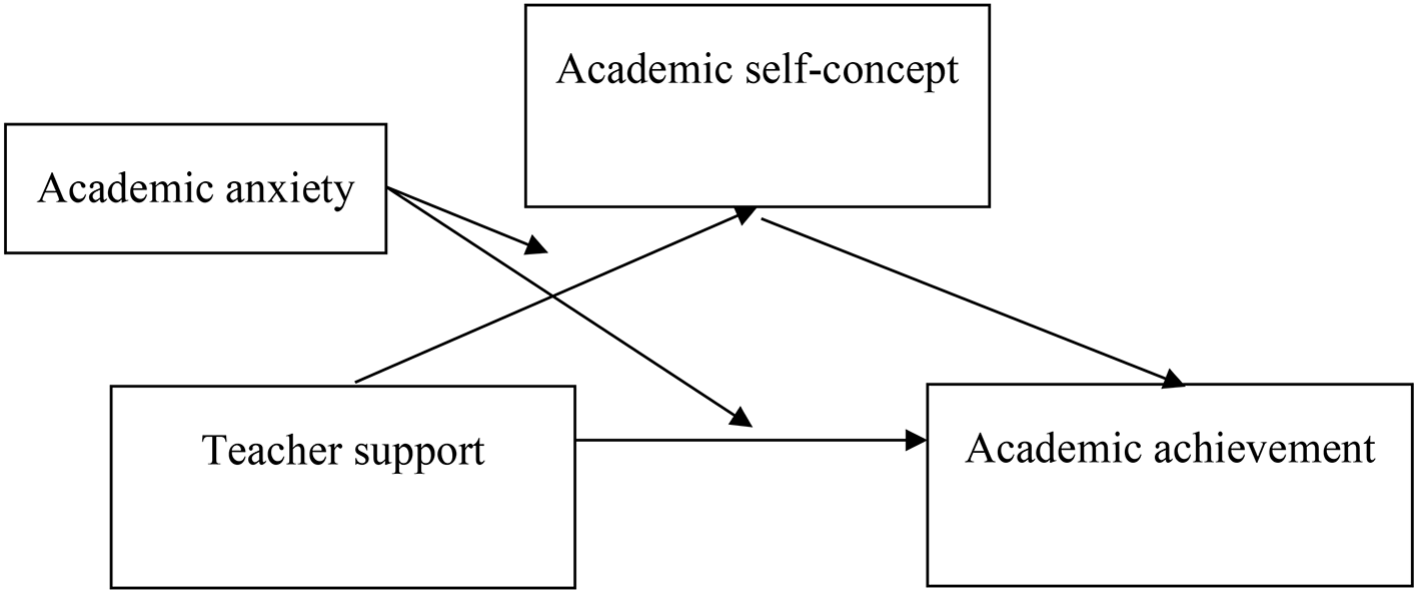
Proposed conceptual model.

We will examine the mechanisms underlying the relationship between teacher support and academic achievement using a moderated mediation model. Specifically, we hypothesize that: (1) academic self-concept (including its four dimensions: perceived academic competence, academic behavioral self-regulation, enjoyment of academic experience, and value of academic achievement) mediates the association between teacher support and academic achievement; (2) academic anxiety moderates the relationships between teacher support and academic self-concept, as well as between teacher support and academic achievement. Specifically, individuals with low academic anxiety will exhibit a stronger positive relationship between teacher support and academic self-concept, as well as academic achievement, whereas those with high academic anxiety will show a weaker positive relationship. The proposed conceptual model, including all hypotheses, is presented in Figure 1.

## 2 Method

### 2.1 Participants and procedure

This study employed a cross-sectional design and recruited boarding junior high school students using convenience sampling. In this study, we selected boarding junior high school students as our research subjects for two primary reasons. Firstly, students from boarding schools spend comparatively less time with their parents and more time with teachers and educators than those from day schools, which could impact the availability of support from these sources (Pfeiffer et al., 2016). It is further inferred that boarding students are likely to receive more support from teachers. Secondly, the complexity of students’ schoolwork has increased sharply since entering middle school, making it challenging for most parents to provide adequate academic support (Geng et al., 2023). However, teachers can offer students the necessary academic tools and guidance (Kim et al., 2017) and significantly influence their academic performance. For instance, Collie et al. (2016) suggested that teacher influence on students’ goals in the classroom is more immediate than that of parents. Moreover, boarding is often a necessary educational pathway for many students due to various reasons, such as living in remote areas, parents working overseas, or choosing education outside their home country (Martin et al., 2021). Therefore, it is crucial to pay more attention to the influence of teacher support on this group. Researchers contacted multiple local junior high schools across regions including Yunnan and Zhejiang provinces, China, for participant recruitment. Inclusion criteria were as follows: (1) be officially enrolled as junior high school students; (2) boarding students are those who stay overnight at school every day except for major holidays; and (3) provide voluntary consent to participate.

A total of 3,000 boarding students were initially recruited. After excluding questionnaires of poor response quality, 2,293 valid responses were retained for analysis, yielding an effective response rate of 76.43%. The participants’ age mean was 13.33 (SD = 0.94). Of the participants, 1,132 (49.40%) were boys, 1,161 (50.60%) were girls. 971 (42.30%) were seventh grade, 868 (37.90%) were eigth grade, and 454 (19.80%) were ninth grade. Participants reported household incomes of less than 2,000 RMB per month (*n* = 823, 35.90%), 2,000–3,000 RMB per month (*n* = 741, 32.30%),3,000–4,000 RMB per month (*n* = 281, 12.30%), 4,000–5,000 RMB per month (*n* = 175, 7.60%), 5,000–6,000 RMB per month (*n* = 116, 5.10%), 6,000–7,000 RMB per month (*n* = 28, 1.20%), 7,000–8,000 RMB per month (*n* = 29, 1.30%), 8,000–9,000 RMB per month (*n* = 31, 1.40%), 9,000–10,000 RMB per month (*n* = 36, 1.60%), more than 10,000 RMB per month (*n* = 33, 1.40%). The Ethics Committee of the author’s organization approved the study.

After receiving training, the researchers visited classes in boarding schools in regions such as Yunnan and Zhejiang to distribute questionnaires from March to June 2024. Prior to the formal distribution, the researchers explained the purpose of the study, the content of the questionnaires, and assured the participants of their rights to anonymity and the option to withdraw at any time. This information was provided to the students, teachers, and their respective parents. After obtaining their consent, we distributed the questionnaires. The questionnaires took a maximum of 10 min to complete.

## 3 Measurements

### 3.1 Teacher support

Teacher support scale compiled by Jia et al. (2009) was adopted in the study. The scale was composed of 7 items, i.e., “I can talk to my teachers about my problems” and “My teachers care about me”. Each item was rated on a four-point scale ranging from 1 (never) to 4 (always). In the current study, all items were averaged to form a composite score, with higher scores indicating higher levels of teacher support. In this study, Cronbach’s *α* coefficient of the scale was 0.78. Confirmatory factor analysis (CFA) showed that the scale fitted the data well (*χ*^2^ = 195.31, df = 13, *χ*^2^/df = 15.02, RMSEA = 0.08, CFI = 0.95, IFI = 0.95, NFI = 0.95, TLI = 0.93, SRMR = 0.04).

### 3.2 Academic self-concept

Academic self-concept questionnaire compiled by Guo et al. (2011) was adopted in the study. The questionnaire was composed of 20 items, including four dimensions: self-control of academic behavior (e.g., “I am extremely diligent in my studies”), value of academic achievement (e.g., “Academic success is of paramount importance to me”), perception of academic ability (e.g., “I possess robust learning abilities”) and experience of academic emotion (e.g., “I derive enjoyment from studying”), which had five items, respectively. Each item was rated on a five-point scale ranging from 1 (strongly disagree) to 5 (strongly agree). In the current study, all items were averaged to form a composite score, with higher scores indicating higher levels of overall academic self-concept or specific dimension of self-concept. In this study, Cronbach’s *α* coefficient of total questionnaire and it’s four dimensions were 0.94, 0.88, 0.89, 0.81, 0.86, respectively. Confirmatory factor analysis (CFA) showed that the scale fitted the data well (*χ*^2^ = 1447.90, df = 166, *χ*^2^/df = 8.72, RMSEA = 0.06, CFI = 0.95, IFI = 0.95, NFI = 0.95, TLI = 0.94, SRMR = 0.05).

### 3.3 Academic achievement

Referring to previous studies on measuring academic achievement (e.g., Sun, 2014), the participants were asked to report their current academic achievement rankings using a five-point scale ranging from 1 (excellent) to 5 (poor). During statistical analysis, reverse scoring was applied, with higher scores indicating better academic achievement. The use of this measure in the present study was mainly based on the following considerations: on the one hand, directly reporting specific academic scores may increase students’ cognitive burden; on the other hand, during the data collection process, we found that some students were reluctant to provide their actual grades, which could affect the completeness of the data.

### 3.4 Academic anxiety

Academic anxiety sub-questionnaire of the academic emotions questionnaire compiled by Dong and Yu (2007) was adopted in the study. The section questionnaire was composed of 7 items, i.e., “Before exams, I experience nervousness and unease” and “I’m quite concerned that my grades may not measure up to others’”. Each item was rated on a five-point scale ranging from 1 (strongly disagree) to 5 (strongly agree). In the current study, all items were averaged to form a composite score, with higher scores indicating higher levels of academic anxiety. In this study, Cronbach’s *α* coefficient of the scale was 0.83. Confirmatory factor analysis (CFA) showed that the scale fitted the data well (*χ*^2^ = 201.72, df = 13, *χ*^2^/df = 15.52, RMSEA = 0.08, CFI = 0.96, IFI = 0.96, NFI = 0.96, TLI = 0.94, SRMR = 0.03).

### 3.5 Data analysis

SPSS 21.0 and AMOS 21.0 was used for the data analysis. Firstly, to showcase the quality of research tools, reliability and validity of scales above mentioned were conducted. Secondly, in order to provide a broad overview of the study, descriptive statistics analysis and correlation analysis between variables were examined. Thirdly, to validate the hypotheses therein, mediation analysis and moderated mediation model were conducted. Specially, Models 4 and 8 of Hayes’ SPSS macro program Process 3.3 (Hayes, 2013) were used in this step. All the continuous variables were averaged. The significance of all effects was tested by setting the bootstrap number to 5,000 and the bias-corrected confidence interval to 95%. The 95% bias-corrected confidence interval do not contain zero, indicating the indirect and moderated mediation effects were significant (Preacher and Hayes, 2008). To get more specific and rich results, academic self-concept were tested at each dimension.

## 4 Results

### 4.1 Preliminary analyses

Table 1 presents the means, standard deviations, and correlations between the variables.

**Table 1:** Descriptive statistics and intercorrelations of variables of interest.

Variables	M	SD	TS	SC	SCBC	SCAV	SCAP	SCEE	AAC	AAN	gender	age	grade	MFI
TS	2.87	0.51	1.00											
SC	3.35	0.70	0.50^***^	1.00										
SCBC	3.10	0.81	0.42^***^	0.90^***^	1.00									
SCAV	4.01	0.86	0.37^***^	0.75^***^	0.52^***^	1.00								
SCAP	3.00	0.81	0.45^***^	0.85^***^	0.76^***^	0.45^***^	1.00							
SCEE	3.30	0.84	0.45^***^	0.88^***^	0.78^***^	0.53^***^	0.67^***^	1.00						
AAC	3.10	1.12	0.16^***^	0.25^***^	0.21^***^	0.23^***^	0.24^***^	0.17^***^	1.00					
AAN	3.75	0.78	0.13^***^	0.18^***^	0.10^***^	0.31^***^	0.04	0.13^***^	0.04	1.00				
gender	1.51	0.50	−0.04	−0.03	−0.05	0.08^***^	−0.11^***^	−0.03	0.14^***^	0.17^***^	1.00			
age	13.33	0.94	−0.02	−0.05^*^	−0.06^**^	−0.01	−0.06^**^	−0.06^**^	−0.01	0.04	−0.02	1.00		
grade	7.77	0.76	−0.03	−0.06^**^	−0.07^**^	0.01	−0.06^**^	−0.09^***^	−0.00	0.03	0.06^**^	0.73^***^	1.00	
MFI	2.49	1.92	0.02	0.01	0.01	−0.02	0.02	0.02	0.07^**^	0.00	0.03	0.02	0.03	1.00

TS, teacher support; SC, academic self-concept; SCBC, self-control of academic behavior; SCAV, value of academic achievement; SCAP, perception of academic ability; SCEE, experience of academic emotion; AAC, academic achievement; AAN, academic anxiety; MFI, monthly family income. *p < 0.05, **p < 0.01, ***p < 0.001.

Apart from the relationship between perception of academic ability of academic self-concept and academic achievement with academic anxiety, all variables examined in this study show significant positive correlations among themselves (all *p* < 0.001). The above significant correlations are valuable, as they lay the foundation for exploring whether academic self-concept and its four dimensions mediate the association between teacher support and academic achievement, as well as whether academic anxiety moderates the relationships between teacher support and academic self-concept, and between teacher support and academic achievement.

In terms of demographic variables, gender demonstrates notable positive correlations with value of academic achievement, academic achievement and academic anxiety, negative correlations with perception of academic ability (all *p* < 0.001). Age and grade are associated significantly negatively with academic self-concept (*p* < 0.05; *p* < 0.01) and its facets—self-control of academic behavior (all *p* < 0.01), perception of academic ability (all *p* < 0.01), experience of academic emotion (*p* < 0.01; *p* < 0.001). And grade is associated significantly positively with gender and age (*p* < 0.01; *p* < 0.001). Moreover, monthly family income shows a significant positive correlation with academic achievement (*p* < 0.01). Because demographic variables exhibit varying degrees of correlation with different study variables, they will be employed as control variables in subsequent analyses.

### 4.2 Mediating role of academic self-concept

**Figure 2:**
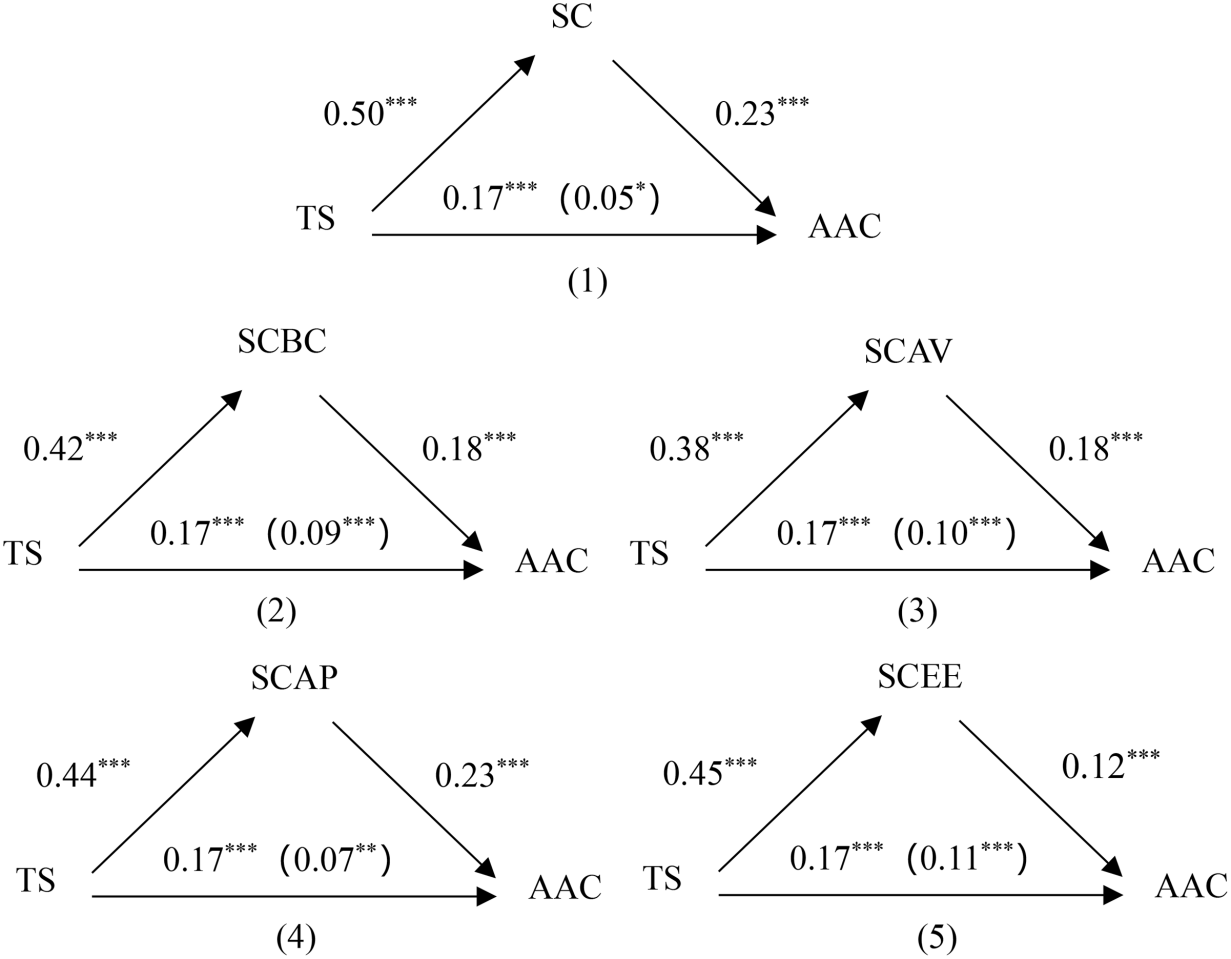
The mediation model. *p < 0.05, **p < 0.01, ***p < 0.001.

In order to test the mediating effect of academic self-concept on the relationship between teacher support and academic achievement under the condition of controlling.

demographic variables, Model 4 of the macro program PROCESS was used. As shown in Figure 2 (1), the results indicated that teacher support had a significant positive effect on academic achievement (total: *β* = 0.17, *t* = 8.23, *p* < 0.001; direct: *β* = 0.05, t = 2.32, *p* < 0.05). Further, teacher support had a significant positive effect on academic self-concept (*β* = 0.50, *t* = 27.61, *p* < 0.001), academic self-concept had a significant positive effect on academic achievement (*β* = 0.23, *t* = 49.76, *p* < 0.001). Therefore, academic self-concept partially mediated the relation between teacher support and academic achievement. The mediation effect accounts for 68% of the total effect, the results were presented in Table 2.

**Table 2:** Total effect, direct effect, and mediation effect of mediating models.

Mediator	Path type	Effect size	BootSE	BootLLCI	BootULCI	Propotion
	Total effect	0.17	0.02	0.13	0.21	
SC	Direct effect	0.05	0.02	0.01	0.10	0.29
	Mediating effect	0.12	0.01	0.09	0.14	0.71
SCBC	Direct effect	0.09	0.02	0.05	0.14	0.53
	Mediating effect	0.08	0.01	0.05	0.09	0.47
SCAV	Direct effect	0.10	0.02	0.06	0.14	0.59
	Mediating effect	0.07	0.01	0.05	0.09	0.41
SCAP	Direct effect	0.07	0.02	0.02	0.11	0.41
	Mediating effect	0.10	0.01	0.08	0.12	0.59
SCEE	Direct effect	0.11	0.02	0.07	0.16	0.65
	Mediating effect	0.06	0.01	0.03	0.08	0.35

To advance the study further, the following section examines whether the four dimensions of academic self-concept, i.e., self-control of academic behavior, value of academic achievement, perception of academic ability and experience of academic emotion, mediate the relationship between teacher support and academic achievement. As shown in Figure 2 (2)–(5), the results indicated that teacher support had a significant positive effect on academic achievement when mediator is self-control of academic behavior (direct: *β* = 0.09, *t* = 4.23, *p* < 0.001), value of academic achievement (direct: *β* = 0.10, *t* = 4.55, *p* < 0.001), perception of academic ability (direct: *β* = 0.07, *t* = 3.01, *p* < 0.01) and experience of academic emotion (direct: *β* = 0.11, *t* = 4.95, *p* < 0.001). Teacher support had a significant positive effect on self-control of academic behavior (*β* = 0.42, *t* = 22.06, *p* < 0.001), value of academic achievement (*β* = 0.38, *t* = 19.51, *p* < 0.001), perception of academic ability (*β* = 0.44, *t* = 23.82, *p* < 0.001) and experience of academic emotion (*β* = 0.45, *t* = 24.20, *p* < 0.001). Academic self-control of academic behavior (*β* = 0.18, *t* = 7.99, *p* < 0.001), value of academic achievement (*β* = 0.18, *t* = 8.41, *p* < 0.001), perception of academic ability (*β* = 0.23, *t* = 10.12, *p* < 0.001) and experience of academic emotion (*β* = 0.12, *t* = 5.39, *p* < 0.001) had a significant positive effect on academic achievement. Therefore, self-control of academic behavior, value of academic achievement, perception of academic ability and experience of academic emotion partially mediated the relation between teacher support and academic achievement. The mediation effect accounts for 47, 41, 59, 35% of the total effect, respectively. The results were presented in Table 2.

Moreover, the 95% confidence intervals for the indirect effect of teacher support on academic achievement through academic self-concept [0.09, 0.14], self-control of academic behavior [0.05, 0.09], value of academic achievement [0.05, 0.09], perception of academic ability [0.08, 0.12] and experience of academic emotion [0.03, 0.08] did not include zero, further indicating the presence of a significant mediating relationship. In sum, the mediation hypothesis, H1, was supported. The results were presented in Table 2.

### 4.3 Moderating role of academic anxiety

**Figure 3:**
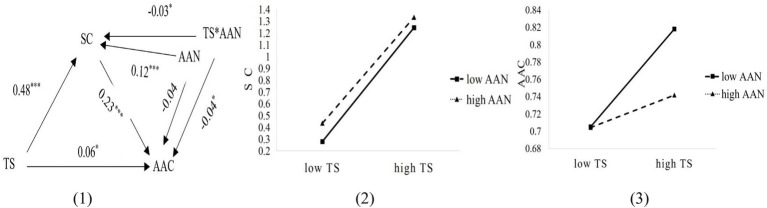
The moderating effect of academic anxiety when mediator is academic self-concept. *p < 0.05, **p < 0.01, ***p < 0.001.

In order to test the moderating effect of academic anxiety on the relationship between teacher support and academic self-concept as well as between teacher support and academic achievement under the condition of controlling demographic variables, Model 8 of the macro program PROCESS was used. As shown in Figure 3 (1), the results indicated that the interaction effect of teacher support and academic anxiety on academic self-concept (*β* = −0.03, *t* = −1.99, *p* < 0.05) and academic achievement (*β* = −0.04, *t* = −1.99 *p* < 0.05) were significant, indicating that academic anxiety moderates the impact of teacher support on academic self-concept and academic achievement. Moreover, the bootstrap method further revealed the interaction effect mentioned above 95%CI are between [−0.07, −0.001] and [−0.07, −0.001], indicating that the moderating influence of academic anxiety is statistically significant in the relationship between teacher support and academic self-concept and then between teacher support and academic achievement. Simple slopes tests indicated that the positive prediction from teacher support to academic self-concept was relatively stronger for students with low levels of academic anxiety (*β* = 0.52, *t* = 20.90, *p* < 0.001) than for students with high levels of academic anxiety (*β* = 0.45, *t* = 18.04, *p* < 0.001), and the positive prediction from teacher support to academic achievement was relatively stronger for students with low levels of academic anxiety (*β* = 0.09, *t* = 3.13, *p* < 0.01) than for students with high levels of academic anxiety (*β* = 0.02, *t* = 0.63, *p* > 0.05), see Figure 3 (2)–(3).

To advance the study further, the following section examines whether academic anxiety had moderating effect when mediator is the four dimensions of academic self-concept, i.e., self-control of academic behavior, value of academic achievement, perception of academic ability and experience of academic emotion. The result indicated that the interaction effect of teacher support and academic anxiety on self-control of academic behavior (*β* = −0.002, *t* = −0.08, *p* > 0.05), perception of academic ability (*β* = 0.002, *t* = 0.11, *p* > 0.05), experience of academic emotion was not significant (*β* = −0.01, *t* = −0.55, *p* > 0.05) were not significant, however, on value of academic achievement (*β* = −0.10, *t* = −5.78. *p* < 0.001) was significant. The interaction effect of teacher support and academic anxiety on academic achievement were significant when mediator is self-control of academic behavior (*β* = −0.05, *t* = −2.37, *p* < 0.05), perception of academic ability (*β* = −0.05, *t* = −2.43, *p* < 0.05) and experience of academic emotion (*β* = −0.04, *t* = −2.31, *p* < 0.05), however, on academic achievement (*β* = −0.03, *t* = −1.33, *p* > 0.05) was not significant.

Moreover, considering the 95% confidence intervals for interaction effect of teacher support and academic anxiety on value of academic achievement [−0.14, −0.07], and the 95% confidence intervals for interaction effect of teacher support and academic anxiety on academic achievement [−0.08, −0.01; all] when mediator is self-control of academic behavior, perception of academic ability and experience of academic emotion, did not include zero, indicating that academic anxiety moderates the impact of teacher support on value of and academic achievement, namely, H2.1 was partly supported and H2.2 was supported.

**Figure 4:**
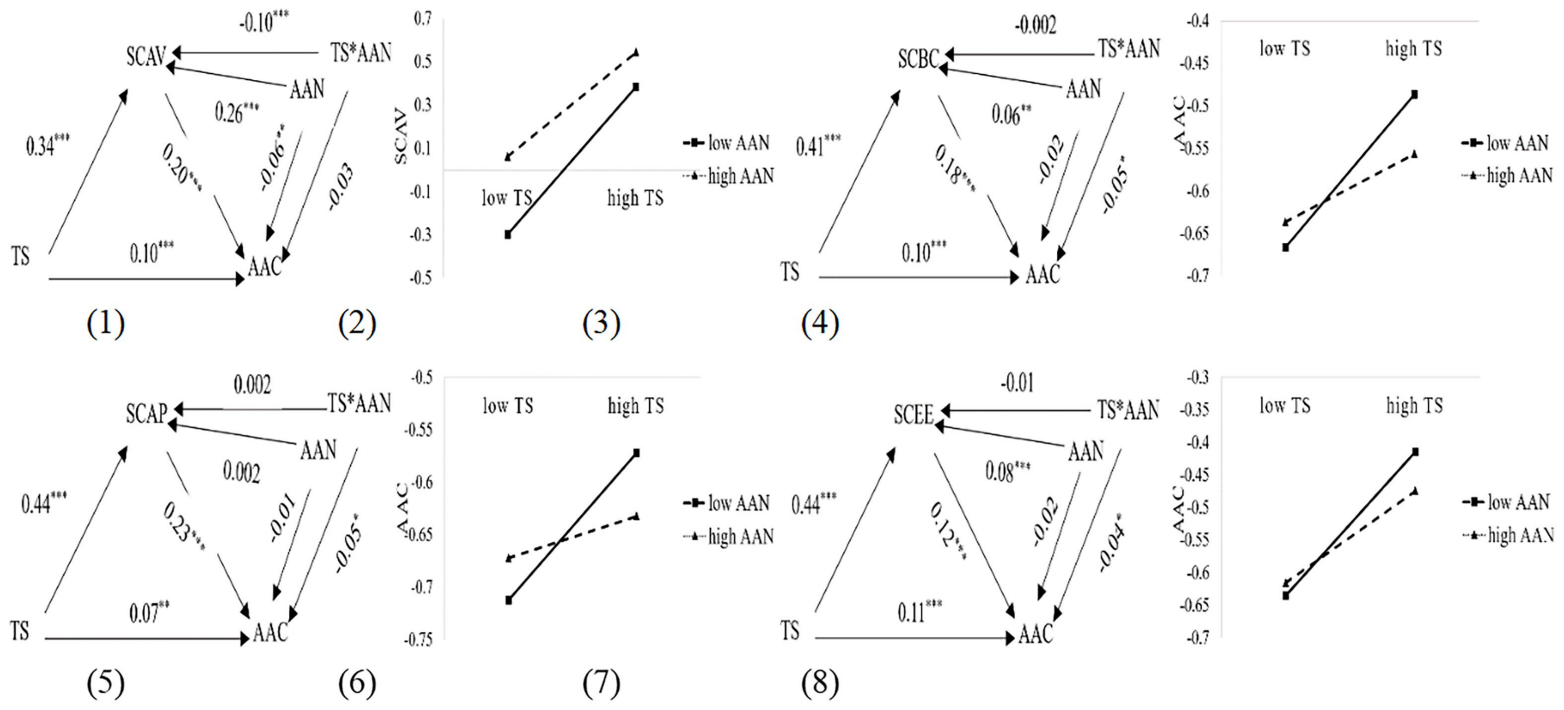
The moderating effect of academic anxiety when mediator is dimensions of academic self-concept. *p < 0.05, **p < 0.01, ***p < 0.001.

To gain a clearer understanding of the moderating role of academic anxiety in the relationship between teacher support and academic self-concept, as well as between teacher support and academic achievement, we followed the practice of relevant literature (Bao et al., 2023) by operationalizing low and high levels of academic anxiety as being one standard deviation below and above the mean, respectively. Simple slopes tests indicated that the positive prediction from teacher support to value of academic achievement was relatively stronger for students with low levels of academic anxiety (*β* = 0.44, *t* = 17.40, *p* < 0.001) than for students with high levels of academic anxiety (*β* = 0.24, *t* = 9.38, *p* < 0.001). See Figure 4 (1)–(2). Moreover, academic anxiety moderates the impact of teacher support on academic achievement when mediator were self-control of academic behavior, perception of academic ability and experience of academic emotion. Simple slopes tests indicated that the positive prediction from teacher support to academic achievement was relatively stronger for students with low levels of academic anxiety (*β* = 0.14, *t* = 4.81, *p* < 0.001; *β* = 0.11, *t* = 3.90, *p* < 0.01; *β* = 0.16, *t* = 5.32, *p* < 0.01) than for students with high levels of academic anxiety (*β* = 0.05, *t* = 1.70, *p* > 0.05; *β* = 0.02, *t* = 0.75, *p* > 0.05; *β* = 0.07, *t* = 2.33, *p* < 0.05) when mediator were self-control of academic behavior, perception of academic ability and experience of academic emotion, respectively. See Figure 4 (3)–(8).

## 5 Discussion

The current study investigated the relationships between teacher support, academic self-concept, academic achievement and academic anxiety. We found that academic self-concept and its four dimensions played mediating roles in the relationship between teacher support and academic achievement. Additionally, academic anxiety moderated the relationship between teacher support and the value of academic achievement within academic self-concept, as well as between teacher support and academic achievement. These noteworthy findings are discussed in detail below.

### 5.1 The mediating role of academic self-concept

The study demonstrated that academic self-concept and its four dimensions partially mediated the relationship between teacher support and academic achievement. In other words, for boarding junior high school students, teacher support promotes their academic self-concept and its dimensions, which in turn improves academic achievement. This finding aligns with previous studies (e.g., Affuso et al., 2022; Tao et al., 2022).

According to social cognitive theory, environmental factors impact external behavior by first influencing internal factors (Bandura, 1997). In this study, teacher support represents the environmental variable, while academic self-concept serves as the internal variable. Thus, it is clear that teacher support can influence boarding students’ academic self-concept, including enhancing their perception of academic competence, behavioral self-regulation, enjoyment of academic experiences, and the value of academic achievement, ultimately leading to improved academic performance. Moreover, as some scholars suggest, academic self-concept is shaped by experiences and reinforced by significant others (Corbière et al., 2006). In China, teachers are often the closest to students and play a significant role in their academic lives (Bao et al., 2023). This is particularly true for boarding students, who often perceive more support from their teachers (Pfeiffer et al., 2016). As students’ awareness of their academic self-concept increases, so do their interest, motivation (Guay et al., 2010; Zhao and Yang, 2022), and ultimately, their academic success (Guo et al., 2011).

### 5.2 The moderating role of academic anxiety

This study demonstrates that academic anxiety moderates the relationship between teacher support and the academic achievement value within the academic self-concept and actual academic achievement of boarding students. To be specific, compared with boarding junior high school students with low academic anxiety, this relationship mentioned above became weaker for boarding junior high school students with high academic anxiety. To summarize, the crucial role of academic anxiety in the model constructed in this study is to act as an attenuator for the aforementioned relationship.

This result can be attributed to the following reasons. Firstly, high anxious boarding students tend to focus more on threatening information (Mathews and MacLeod, 1994), meaning they are more likely to concentrate on negative feedback or slightly strict language from teachers while overlooking other positive aspects. This selective attention diminishes the effectiveness of teacher advice and weakens their recognition of the value of academic achievement and the likelihood of achieving higher academic achievement. In the boarding environment, the lack of daily family support may exacerbate these tendencies among highly anxious students, further impacting their academic performance. However, low-anxiety boarding students are more likely to correctly interpret teacher suggestions, thereby better utilizing these recommendations to enhance their academic performance and understanding of the importance of academic achievement. Secondly, emotion regulation theory (Gross, 2002) suggests that highly anxious boarding students, due to the unique pressures of the boarding environment, are more prone to excessive emotional reactions when receiving teacher feedback, leading to distraction and reduced focus on learning, thereby affecting academic performance. Meanwhile, cognitive resource theory (Eysenck et al., 2007) also indicates that highly anxious boarding students expend significant cognitive resources managing their anxiety, leaving them less equipped to handle complex academic tasks, further diminishing their academic performance. These theories collectively explain why academic anxiety weakens the positive impact of teacher support on the academic achievement of boarding students, particularly among those with high anxiety. In sum, low-anxiety boarding students are better able to manage their emotions, adopt positive coping mechanisms, and thereby fully utilize teacher support, leading to improved academic performance and a stronger recognition of the value of academic achievement. Conversely, highly anxious boarding students, due to insufficient emotion regulation and cognitive resources, struggle to benefit from teacher support, resulting in poorer academic performance.

In summary, this study demonstrates that teacher support positively influences academic achievement, partly through academic self-concept, and that academic anxiety moderates these relationships. By integrating mediating and moderating mechanisms within a single framework, the study fills gaps in previous research and provides a more comprehensive understanding of how and under what conditions teacher support affects academic outcomes among boarding junior high school students. These findings also offer theoretical and practical insights, emphasizing the importance of addressing students’ academic anxiety in designing effective support strategies.

### 5.3 Limitations

This study has several limitations that should be noted. First, the cross-sectional design does not allow for establishing causal relationships between variables. Caution is required when interpreting the results, as variables like anxiety and academic achievement (e.g., David et al., 2020) are interrelated are found in some studies. Future longitudinal research is needed to clarify the direction of these effects. Second, the study was conducted in boarding schools in a few provinces in China and employed a convenience sampling method, which may introduce certain biases and potentially limit the generalizability of the findings. Future research should aim to include larger and more diverse participant pools to further validate the applicability of the results. Third, in the confirmatory factor analysis of teacher support, academic self-concept, and academic anxiety, although indices such as RMSEA, CFI, IFI, NFI, TLI, and SRMR were within acceptable ranges, the χ^2^/df values were all greater than 8. This may adversely affect the model, suggesting that future research should further analyze and optimize the appropriateness of the aforementioned measurement tools. Fourth, academic achievement was measured by students’ self-reported class rank, which, while easy to collect, may raise concerns about accuracy and objectivity. Future research should incorporate both subjective and objective measures of academic achievement.

### 5.4 Implications

The results inspired us to improve boarding junior high school students’ academic achievement from the following aspects.

Firstly, attach importance to the degree of teacher support. Teachers should cultivate an awareness of providing support to boarding junior high school students, including strive to establish relationships of mutual trust and respect with them, actively listen to their concerns, and provide targeted assistance. Schools should play the roles of facilitator and supervisor in this process. As facilitators, they should provide training to enhance teachers’ understanding of student issues and improve their ability to effectively serve students. As supervisors, they should establish reward and disciplinary mechanisms based on the extent to which teachers support boarding junior high school students. This includes rewarding or penalizing teachers according to the level of support they provide to boarding junior high school students.

Secondly, improve the cultivation of academic self-concept. Schools should establish regular mechanisms for understanding and addressing boarding junior high school students’ academic concerns, helping them identify and resolve academic issues early to enhance their confidence and improve their academic self-concept. Teachers should provide timely and appropriate feedback on students’ academic progress to help them accurately assess their learning status, feel their own progress, and strengthen their motivation and self-evaluation in learning. Parents should also create a positive and supportive learning environment, guiding and encouraging students to develop positive self-assessment in their academic pursuits.

Finally, pay more attention to boarding junior high school students’ academic anxiety status, especially, students with high academic anxiety should be concerned. Because academic anxiety was found to be a attenuate factor for boarding junior high school students to cope with academic self-concept and achievement in this study. For these students with high academic anxiety, schools, teachers and parents should help students understand the negative impact of high anxiety on their own health and academic performance, listen to their various difficulties and concerns regarding their studies, understand potential influencing factors, and provide psychological counseling techniques or other forms of support to help students build adaptive responses toward failure, alleviate academic anxiety and foster a positive attitude towards their studies.

## 6 Conclusion

This study contributes to the existing literature by delving into the intricate relationships between teacher support, academic achievement, academic self-concept, and academic anxiety, particularly within the context of boarding junior high school students. It highlights the mediating role of academic self-concept in linking teacher support to academic achievement, while also uncovering the moderating effect of academic anxiety on these relationships. The findings emphasize the importance of addressing both self-concept and anxiety in educational strategies to enhance academic outcomes. Furthermore, the study proposes targeted interventions aimed at improving the academic performance of boarding students, thereby offering practical insights for educators and policymakers.

## Data Availability

The raw data supporting the conclusions of this article will be made available by the authors, without undue reservation.
